# Energy imbalance in oPOI ovarian granulosa cells is linked to reduced glucose bioavailability and metabolism

**DOI:** 10.1186/s12958-025-01426-8

**Published:** 2025-07-03

**Authors:** Weronika Marynowicz, Paulina Głód, Dawid Maduzia, Justyna Gogola-Mruk, Anna Ptak

**Affiliations:** 1 Laboratory of Physiology and Toxicology of Reproduction, Faculty of Biology, Institute of Zoology and Biomedical Research, Jagiellonian University, Cracow, Poland; 2 Doctoral School of Exact and Natural Sciences, Jagiellonian University, Cracow, Poland; 3 Infertility Treatment Centre PARENS, Cracow, Poland

**Keywords:** Occult premature ovarian insufficiency (oPOI), Granulosa cells, Mitochondrial dysfunction, Energy metabolism, Glucose transporters

## Abstract

**Background:**

Premature ovarian insufficiency (POI), affecting approximately 1% of women under 40, is associated with impaired fertility. Occult POI (oPOI), an initiating form, is more challenging to detect but still allows potential success with IVF. Recent studies suggest a possible link between granulosa cell (GC) mitochondrial dysfunction and POI, as mitochondria are critical for energy production and reproductive function.

**Methods:**

We recruited 81 women undergoing IVF which included: 25 women with oPOI defined as a low anti-Müllerian hormone (AMH) level (≤ 1.1 ng/mL) and under the age of 40 without raised follicle-stimulating hormone (FSH) levels and 56 healthy women (male or tubal factor infertility). Molecular analysis of GCs and CCs involved RT-qPCR and functional assays, including Seahorse metabolic profiling, fluorometric/luminescent enzyme activity tests, and mitochondrial fluorescent staining.

**Results:**

We found that cumulus cells (CCs) from oPOI women showed reduced energy capacity. Similarly, GCs shifted toward glycolysis in oPOI, leading to lower ATP production. Despite similar glucose levels in FF between groups, oPOI CCs exhibited impaired glucose uptake and metabolism, with decreased GLUT1 and reduced hexokinase 2 (HK2) activity. In GCs, reduced GLUT1 but increased HK2 gene expression suggests compensatory metabolic reprogramming to maintain energy balance through enhanced glycolysis. Additionally, oPOI women had a lower level of estradiol, despite having a normal FSH level and a decreased estradiol/oocyte count.

**Conclusions:**

This study indicated that in the case of oPOI, disruption may extend beyond the ovaries to impact the entire HPO axis. Furthermore, reduction of ATP production is connected with lower glucose uptake and may have implications for fertility in oPOI patients. It also highlights the potential for therapeutic strategies focused on improving glucose metabolism and mitochondrial biogenesis.

## Introduction

Premature ovarian insufficiency (POI), a condition characterized by ovarian dysfunction before the age of 40, has been acknowledged since 1942 [1] under various terminologies and definitions, such as primary ovarian insufficiency (POI), premature ovarian failure (POF), and early menopause. The prevalence of POI is estimated to be approximately 1 in 100 women under 40 years old [2] posing significant challenges for fertility treatments, especially as the trend of delayed childbearing continues in developed countries.

Infertility may be the earliest manifestation of POI [3]. According to different FSH levels, fecundity, and menstrual status, POI has been subdivided into progressive stages [3]. Patients with evident POI exhibit amenorrhea and follicle-stimulating hormone (FSH) levels above 25 IU/L [4], were previously referred to as POF. Moreover, fertility treatments, such as in vitro fertilization (IVF), are unsuccessful in women with evident POI, as the ovaries tend to be unresponsive to hormone stimulation. However, about 50% of patients will have varying degrees of residual ovarian function with periods characterized by oligomenorrhea and spontaneous ovulations. It is estimated that approximately 5–10% of patients with POI can conceive spontaneously [5]. It seems that most of these patients have early stages of POI (occult form, oPOI) with low ovarian reserve. Occult ovarian failure was first described by Cameron and colleagues in 1988 as the triad of infertility, regular menses, and elevated plasma FSH concentration [6]. In 2009, Streuli et al., described oPOI as partial ovarian insufficiency in women under the age of 40, including infertility, slightly raised FSH levels, low levels of anti-Müllerian hormone (AMH), and/or resistance to ovarian stimulation in women with either regular or irregular cycles [7]. In 2017, Guzel et al., defined oPOI as a serum AMH level ≤ 1.1 ng/mL (a marker of diminished ovarian reserve (DOR)) according to one of the Bologna criteria [8]. In patients with oPOI, spontaneous pregnancy and IVF programs with their own oocytes are still possible. Current literature on screening for oPOI in women is sparse, emphasizing the need for research that characterizes the condition specifically at the cellular level.

Recent studies have reported an association between mitochondrial diseases and evident POI in women [9]. Furthermore, data suggests that mitochondrial dysfunction may play a role in POI pathogenesis, underscoring the complex interplay between cellular energetics and reproductive health [10]. The mitochondria serve as the principal site of cellular energy production, primarily through the oxidative metabolism of glucose, fatty acids, and amino acids via the tricarboxylic acid (TCA) cycle and oxidative phosphorylation (OXPHOS) [11]. Although glycolysis, occurring in the cytosol, contributes to ATP synthesis through the anaerobic breakdown of glucose to pyruvate, the majority of cellular ATP is generated through mitochondrial oxidative pathways. Glucose is essential for energy production by cumulus cells (CCs), and supply of pyruvate to the oocyte for ATP production. There is bidirectional communication between the oocyte and the CCs, which is essential for oocyte competence and proper embryogenesis [12]. Moreover, CCs and granulosa cells (GCs) are connected by gap junctions, allowing the transfer of metabolites and regulatory factors between the entire follicle compartment and the oocyte [13]. GCs and CCs dynamically regulate energetic metabolism, including glycolysis and oxidative phosphorylation (OXPHOS). Therefore, mitochondrial dysfunction may perturb metabolite trafficking leading to poor oocyte quality, ovarian aging, and infertility [14]. Importantly, recent research suggests that mitochondrial dysfunction in GCs may play a role in the pathogenesis of POI [15].

Analysis of GCs and CCs is considered one of the best methods for assessing oocyte viability as a non-invasive strategy [16]. However, the specific energy production profile of CCs and GCs from oPOI patients has not been described before. A better understanding of the role of mitochondria in oPOI may improve the management of women with oPOI and treatment methods in the future. It may also provide a promising new avenue for oPOI research. This study aims to describe the differences in energy metabolism in GCs and CCs from healthy and oPOI patients with a strong focus on mitochondrial functions elucidating the molecular complexity of energy metabolism in ovarian follicle.

## Materials and methods

### Patients

The study included 81 women who had undergone IVF in the Fertility Clinic. Patients before the age of 40 were allocated into two groups: a control (healthy) group consisting of 56 women with male factor infertility or a blocked fallopian tube diagnosis and an oPOI group consisting of 25 infertile women with recognized, unexplained infertility and an AMH level of < 1.1 ng/mL (Table 1.). Infertile women over the age of 40, with polycystic ovarian syndrome, endometriosis, a history of ovarian or pelvic surgery, radiotherapy, chemotherapy, autoimmune diseases, recurrent abortion, or any chronic disease were excluded from the study. The good blastocyst rate (GBR) was calculated as GQB divided by the number of MII oocytes for ICSI, multiplied by 100%. The clinical pregnancy rate (CPR) was calculated as the number of clinical pregnancies divided by the number of transfers, multiplied by 100%.

**Table 1:** Clinical and embryological characteristics of patients with oPOI and healthy women

	Healthy (*n* = 56)	SD	oPOI (*n* = 25)	SD	*p* value	
**Age**	**32.23**	3.42	**32.20**	4.45	0.9746	**ns**
**BMI** [kg/m^2^]	**23.56**	5.67	**23.35**	3.07	0.7954	**ns**
**AMH** [ng/ml]	**3.83**	2.87	**0.83**	0.54	< 0.0001	***
**FSH** [mIU/ml]	**6.11**	3.17	**7.12**	2.50	0.171	**ns**
**E**_**2**_ [pg/ml]	**2483.91**	1513.15	**949.72**	545.83	< 0.0001	***
**E**_**2**_ **/Oocyte**	**190.24**	82.00	**152.37**	54.83	0.0265	*
**AFC**	**12.88**	5.87	**6.09**	2.24	< 0.0001	***
**RO**	**14.47**	8.05	**5.80**	2.78	< 0.0001	***
%of AFC	112%		95%			
**MII**	**9.91**	7.01	**3.96**	2.75	< 0.0001	***
% of RO	68%		68%			
**MII for ICSI**	**8.44**	5.52	**3.83**	2.57	< 0.0001	***
% of RO	58%		66%			
% of MII	85%		97%			
**MI for ICSI**	**0.51**	1.77	**0.04**	0.20	0.0579	**ns**
% of RO	4%		0.7%			
**Degenerate**	**1.62**	3.33	**0.76**	0.97	0.0837	**ns**
%of RO	11%		13%			
**GQB**	2.07	1.67	0.76	0.91	< 0.0001	***
**GB rate**	41%		26%			
**CP rate**	62%		53%			

### Human sample collection and culture

All samples were brought to the laboratory within 1 h. Cumulus cells were collected following oocyte retrieval. Follicular fluid (FF) samples were obtained from pooled follicular aspirates of follicles. Mural GCs were retrieved using separation solution (Lymphosep, L0560, Biowest, Nuaille, France). Follicular Fluid was collected and then frozen in -80 °C for future analysis. CCs and GCs cells were resuspended in DMEM/F-12 (21041025, ThermoFisher Scientific, Waltham, MA, USA) cell culture media containing 5% FBS (Fetal Bovine Serum, S181H-500, Biowest, Nuaille, France), counted, and then seeded into flat 96-well plates or XFp cell culture microplates under standard conditions. All analyses were performed within 24 h.

### Assessment of bioenergetic parameters

Characterization of the bioenergetic properties of GCs and CCs was performed using a Seahorse XFp Extracellular Flux Analyser (Agilent Technologies, Santa Clara, CA, USA). The Seahorse XFp Real-Time ATP Rate Assay Kit (103591-100, Agilent Technologies) was used to determine the bioenergetics parameters according to manufacturer’s instructions. Basal OCR was determined by performing three measurements before the addition of the metabolic modulators. Raw data were analysed using Seahorse Analytics (Version: 1.0.0-699) with graphical presentation using Graphpad Prism version 6.00. Data normalisation: Protein concentration in each well was determined using RIPA Lysis Buffer (#89900, ThermoFisher Scientific) with protease inhibitor (#1862209, ThermoFisher Scientific) according to manufacturer’s protocol. The protein concentration was determined using a Nanodrop spectrophotometer (#DS-11 DeNovix, Wilmington, DE, USA).

### Mitochondrial membrane potential measurement (JC-1) and active mitochondria staining (Mitotracker Red)

Mitochondrial membrane potential in CCs and GCs was measured using the JC-1 fluorescence dye (Sigma-Aldrich, Saint Louis, MO, USA). Staining was performed within 24 h of cell culture. JC-1 (10 µg/mL) was incubated for 10 min under standard conditions. The red/green fluorescence ratio was calculated and used as an indicator of mitochondrial membrane potential (ΔΨm) as a universal indicator of mitochondrial health. The quantity of active mitochondria was measured using 500 nM Mitotracker Deep Red (ThermoFisher Scientific) under standard conditions. Fluorescence intensity was measured using the VarioscanTM LUX Multimode Microplate Reader (ThermoFisher Scientific). Images were obtained using an Axiocam 503 Bright Field/Fluorescence Microscope (Carl Zeiss, GmBH) and were analysed using ImageJ.

### Gene expression analysis

Total RNA isolation and cDNA synthesis were performed using the TaqMan Gene Expression Cells-to-CT Kit (Applied Biosystems, ThermoFisher Scientific) according to the manufacturer’s instructions. The expression of hexokinase 2, HK2 (Hs00606086_m1), isocitrate dehydrogenase (NADP(+)) 2, IDH2 (Hs00953879_m1), glucose transporter 1 (GLUT1, SLC2A1; Hs00892681_m1), glucose transporter 4 (GLUT4; SLC2A4; Hs00168966_m1), solute carrier family 5 member 1, SLC5A1 (Hs01573793_m1), and solute carrier family 5 member 2, SLC5A2 (Hs00894642_m1) was measured by real-time qPCR using a TaqMan Gene Expression Assay (Applied Biosystems, ThermoFisher Scientific). Expression levels were normalized to that of the 18s gene (4310893E). Relative expression was quantified using the 2^−ΔΔCt^ method.

### Glucose level in FF

Glucose concentrations were determined in follicular fluid samples from all patients. Samples were cleared of blood and frozen at -80 °C until analysis. Glucose [ng/mL] was measured using the Glucose Colorimetric Detection Kit (EIAGLUC, Thermo Fisher Scientific) according to the manufacturer’s instructions. Absorbance was measured using the VarioscanTM LUX Multimode Microplate Reader (ThermoFisher Scientific).

### Hexokinase activity

CCs and GCs cells were collected after 24 h of cell culture. Hexokinase activity was measured using an Assay Kit (ab136957, abcam) according to the manufacturer’s instructions. Fluorescence was measured using the VarioscanTM LUX Multimode Microplate Reader (ThermoFisher Scientific).

### Glucose uptake

The uptake of the glucose was measured by the Glucose Uptake-Glo Assay (J1341, Promega, Madison, WI, USA), according to the manufacturer’s instructions. The cells were incubated for 1 h. Luminescence was measured using the VarioscanTM LUX Multimode Microplate Reader (ThermoFisher Scientific).

### Statistical analysis

Statistical analyses were performed using GraphPad Software (La Jolla, CA, USA). The values are presented as mean ± standard deviation (SD). Significant differences were tested using parametric Student’s t-test with Welch’s correction or two-way ANOVA, followed by Tukey’s/ Sidak’s test. The level of significance was set at *p* < 0.05.

## Results

### Characterization of the clinical data of healthy and oPOI groups

We found significantly lower AMH (0.83 vs. 3.83 ng/mL of healthy women; *p* < 0.0001), and estradiol (949.72 vs. 2483.91 pg/mL of healthy women; *p* < 0.0001) levels in oPOI women. The estradiol per oocyte count rate was also lower decreased among oPOI women (152.37 vs. 190.24 of heathy women; *p* = 0.0265). However, there were no significant differences in age, BMI, or FSH levels between the two groups The analysis of embryological data represent a statistically significant decrease in the antral follicle count (AFC) of oPOI women (6.09 vs. 12.88 of healthy women). Moreover, the number of total retrieved oocytes (RO), with the number of MII oocytes and MII used for ICSI was also reduced in the oPOI group, comparing to healthy women. The number of good-quality blastocysts (GQB) was determined using the Gardner blastocyst grading system. The GQB of the retrieved blastocysts from the healthy group was 2.07 ± 1.7, whereas in the oPOI group it was 0.76 ± 0.91 (*p* < 0.0001). The GQB rate was 41% versus 26% for oPOI women. The pregnancy rate (clinical pregnancy per all ICSI cases, CP rate) was 62% in the healthy group versus 53% in the oPOI group (Table 1).

### Increased ΔΨm in GCs and CCs of oPOI compared with healthy women

**Fig. 1:**
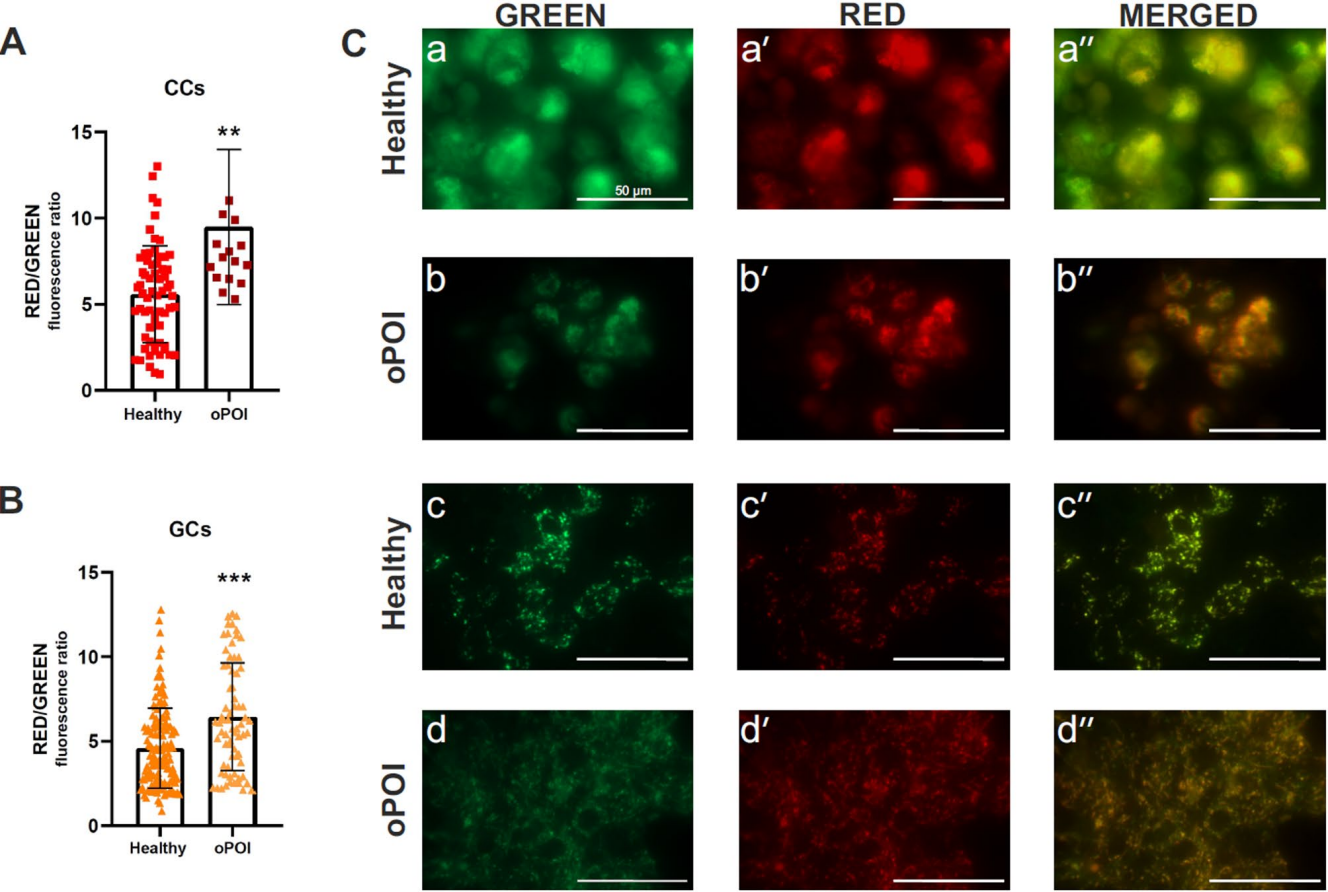
Mitochondrial membrane potential (MMP, ΔΨm) of cumulus cells, CCs (**A**) and granulosa cells, GCs (**B**) from healthy and oPOI women measured by red/green JC-1 fluorescence intensity ratio. (**C**) Visualization of active (red, JC aggregates) and inactive (green, JC aggregates) mitochondria of CCs (a-a'' and b-b'') and GCs (c-c'' and d-d''). Merged photos show the dominant color. Error bars denote the means ± standard deviations (SD). Student’s t-test (**p < 0.01, ***p < 0.001)

Changes in mitochondria function have been postulated to be one of the critical factors in the development of POI. We measured ΔΨm using the red/green JC-1 fluorescence intensity ratio. We observed a statistically significantly higher red/green ratio in the CCs and GCs of oPOI women than in those of healthy women (Fig. 1A-B). Visualization of JC aggregates showed intense yellow fluorescence in merged pictures, confirming that ΔΨm was increased in POI cells (Fig. 1C).

### Change in the bioenergetic profile of oPOI women

**Fig. 2:**
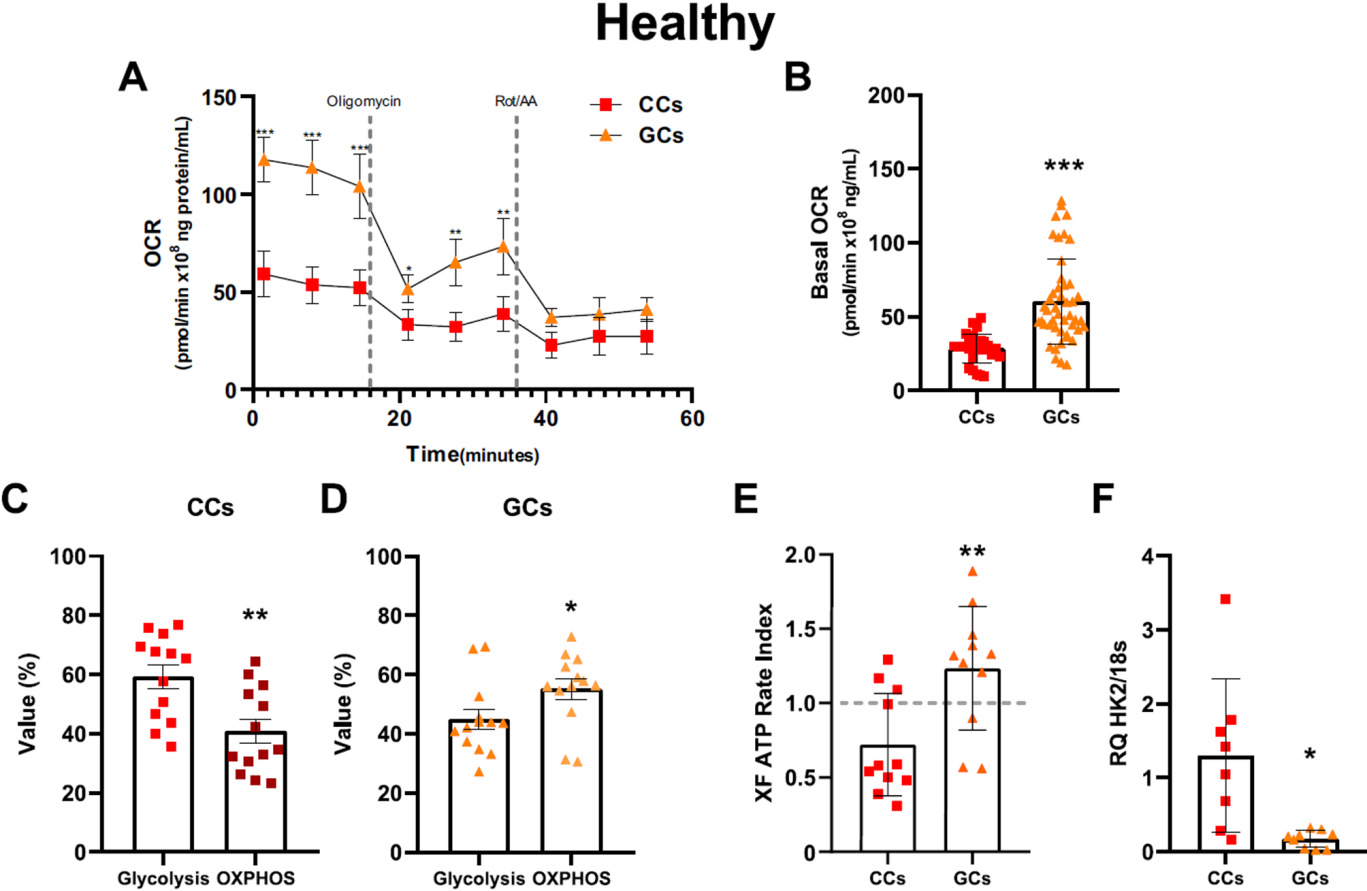
Characterization of the bioenergetic phenotypes of healthy women. (**A**) Representative OCR data plot of the results of the XF ATP Rate Assay. (**B**) Basal Oxygen Consumption Rate in cumulus cells (CCs) and granulosa cells (GCs). (**C-D**) The dominant process for ATP production in CCs and GCs. (**E**) XF ATP Rate Index in CCs and GCs. (**F**) Expression of hexokinase II (HK2), the enzyme involved in the first step of glycolysis, Each data point is the average of at least three independent measurements. Error bars denote the means ± standard deviations (SD). Statistical analyses were performed by 2-way ANOVA with Sidak’s test and by t-test (*p < 0.05, **p < 0.01, ***p < 0.001, ns, no significances)

Our finding that Δψm is altered in oPOI women prompted us to examine the metabolic profiles of GCs from healthy and oPOI women. We observed that in healthy women, CCs consumed less oxygen compared to GCs (Fig. 2A, B, *p* < 0.001) and mainly used glycolysis for ATP production (Fig. 2C, *p* < 0.01). By contrast, in GCs, ATP was mainly derived from OXPHOS (Fig. 2D, *p* < 0.05), and the oxygen consumption rate was significantly higher compared to CCs (Fig. 2A, B, *p* < 0.001). An XF ATP Rate Index greater than 1 for GCs indicates that more than 50% of cellular ATP was produced by OXPHOS, whereas in CCs, more than 50% of total ATP was derived from glycolysis (Fig. 2E, *p* < 0.01). Finally, RT-qPCR results showed that HK2 expression was higher in CCs than in GCs (Fig. 2F, *p* < 0.05), which is consistent with our results.

**Fig. 3:**
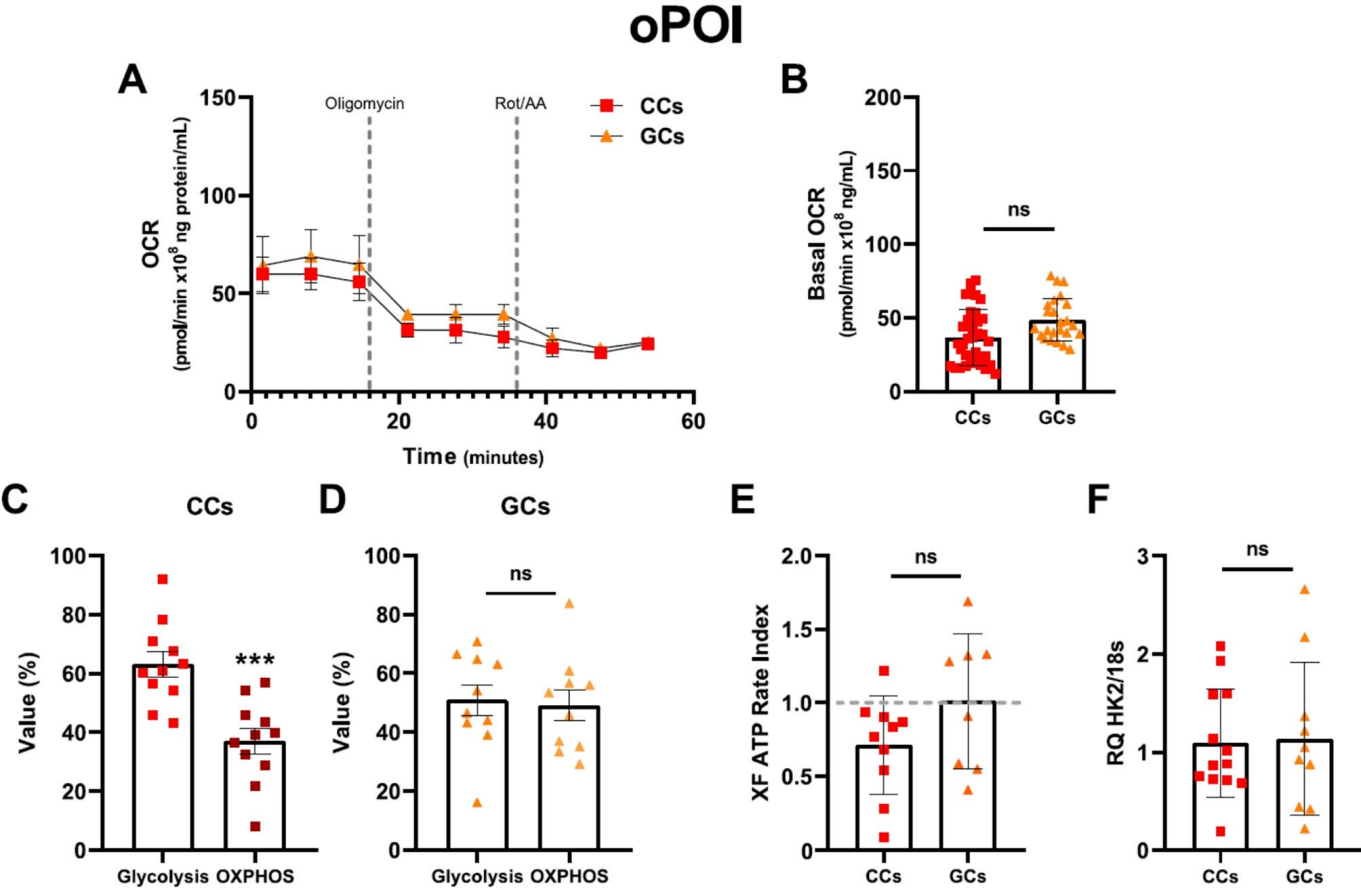
Characterisation of the bioenergetic phenotypes of oPOI women. (**A**) Representative OCR data plot of the results of the XF ATP Rate Assay. (**B**) Basal Oxygen Consumption Rate in cumulus cells (CCs) and granulosa cells (GCs). (**C-D**) The dominant process for ATP production in CCs and GCs. (**E**) XF ATP Rate Index in CCs and GCs. (**F**) Expression of hexokinase II (HK2), the enzyme involved in the first step of glycolysis. Each data point is the average of at least three independent measurements. Error bars denote the means ± standard deviations (SD). Error bars denote the means ± standard deviations (SD). Statistical analyses were performed by 2-way ANOVA with Sidak’s test and by t-test (***p < 0.001, ns, no significances)

In oPOI women, OCR was at similar average level in CCs compared to GCs (Fig. 3A, B). Moreover, we did not observe differences in the XF ATP Rate Index between CCs and GCs (Fig. 3C), indicating an altered metabolic phenotype of these cells compared with healthy women. Indeed, GCs produced equal amounts of ATP from both OXPHOS and glycolysis (Fig. 2E), while CCs used glycolysis mainly for ATP production (Fig. 3D, *p* < 0.001). However, the expression of the glycolytic gene HK2 was similar in both cells (Fig. 3F).

### Total ATP production in oPOI patients was reduced

**Fig. 4:**
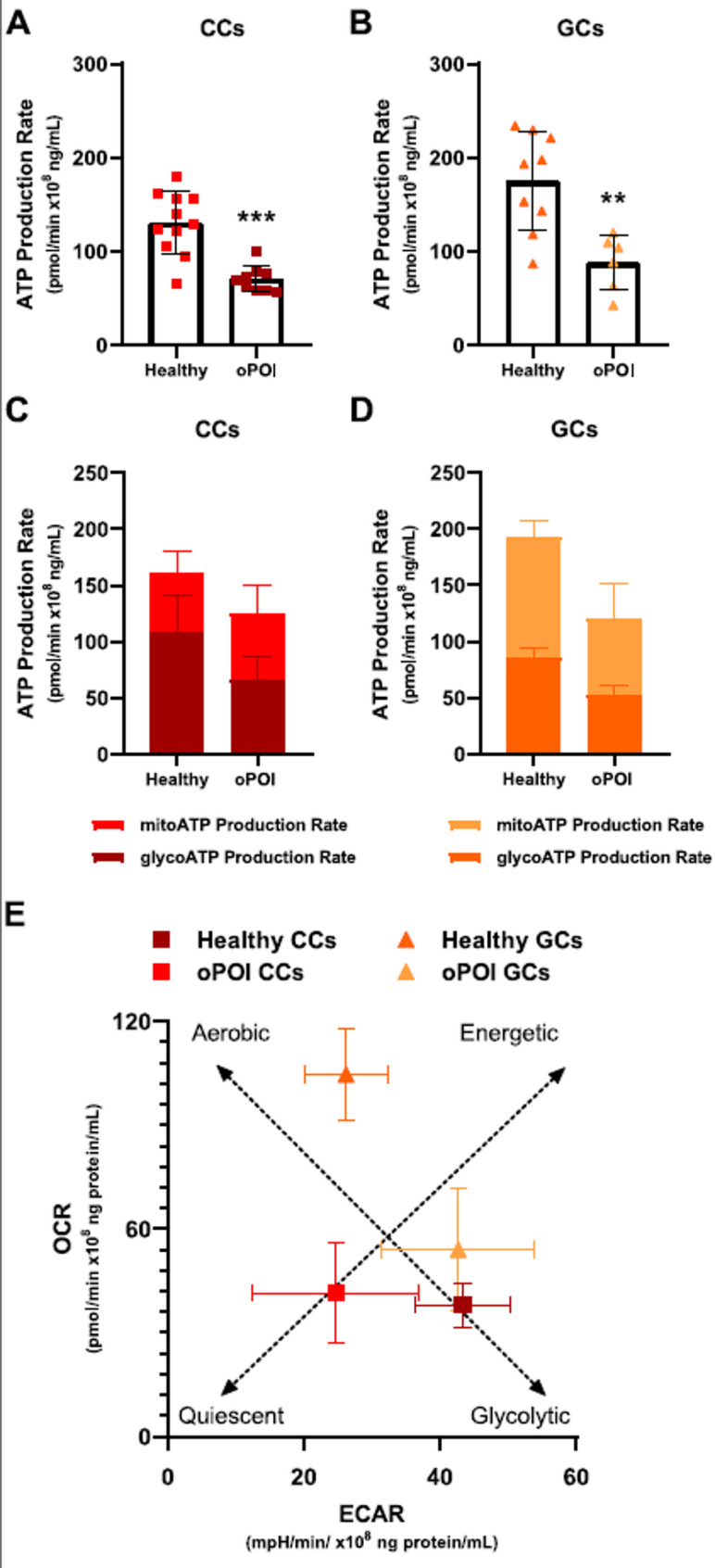
ATP production rates of healthy women and oPOI women. Total ATP production in cumulus cells (CCs) (**A**) and granulosa cells (GCs) (**B**) from healthy and oPOI women. Representative plots of the ATP production rate in CCs (**C**) and GCs (**D**) from healthy and oPOI women measured using the XF ATP Rate Assay. Energy map for CCs and GCs (**E**) from healthy and oPOI women. Dotted lines represent the aerobic-glycolytic axis and the quiescent-energetic axis. Each data point is the average of at least three independent measurements. Error bars denote the means ± standard deviations (SD). Statistical analyses were performed by t-test. (**p < 0.01, ***p < 0.001)

Next, we analyzed the total ATP production rates of CCs and GCs, which showed that GCs were more energetic than CCs (130.387 vs. 70.266 pmol/min x10^8^ ng/mL in CCs of healthy women; *p* < 0.001 and 175.170 vs. 87.982 pmol/min x10^8^ ng/mL in GCs of healthy women; *p* < 0.01 (Fig. 4A, B). The total ATP production rates of CCs and GCs from oPOI women were decreased (Fig. 4C-D), which was mainly contributed to the glycolytic pathway in CCs (Fig. 4C) and the mitochondrial pathway in GCs (Fig. 4D). The energy map shows that CCs from oPOI women shift towards less energetic while GCs from oPOI women are more glycolytic (Fig. 4E).

### Altered glucose transporter expression and HK2 activity in oPOI patients

**Fig. 5:**
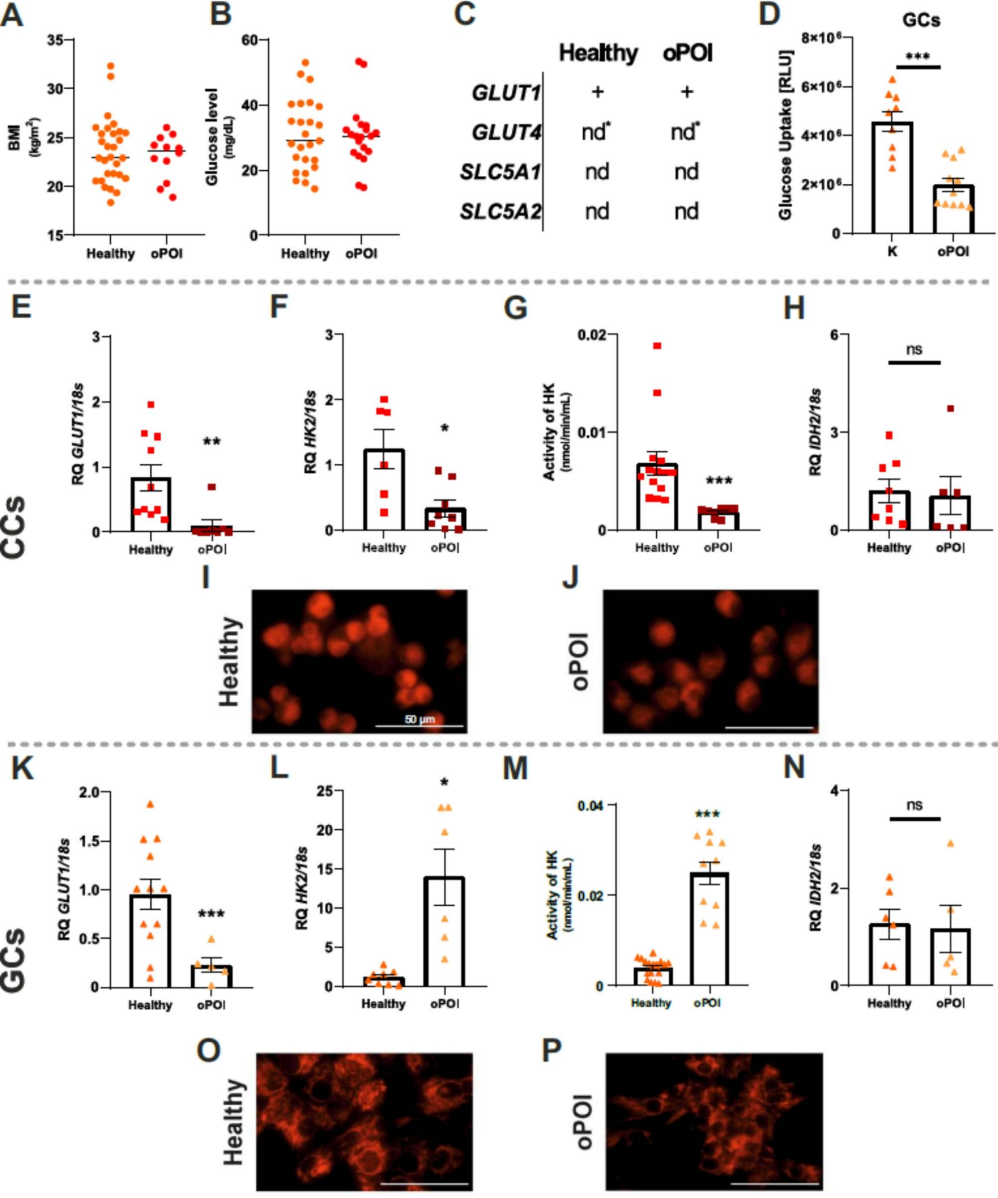
BMI and follicular fluid glucose levels with the expression of metabolic-related genes. (**A**) BMIs of healthy and oPOI women. (**B**) Glucose concentration in follicular fluid and (**C**) transporter gene expression in cumulus cells (CCs) and granulosa cells (GCs) of healthy and oPOI women. Nd, no detected. nd*, not detected in almost all cases. Glucose uptake in GCs (**D**). Cumulus cells, CCs (**E**) or Granulosa Cells, GCs (**K**): Glucose transporter 1 (GLUT1) and CCs (**F**) or GCs (**L**) hexokinase II (HK2) expression. CCs (**G**) or GCs (**M**) HK activity, and CCs (**H**) or GCs (N) NADP-dependent isocitrate dehydrogenase 2 (IDH2) expression. CCs (**I, J**) or GCs (O, P) mitochondrial activity image analysis. Data for each sample are expressed as mean ± SD. Statistical analyses were performed by t-test (*p < 0.05, **p < 0.01, ***p < 0.001)

To further characterize the metabolic changes in the CCs and GCs of oPOI patients, we first compared BMI and glucose levels in FF, but did not find significant differences between oPOI and healthy women (Fig. 5A, B). GLUT1 gene expression was detected in GCs from Healthy and oPOI. GLUT4 gene expression was not detected in all samples, the obtained data were not statistically significant. We did not detect SLC5A1 and SLC5A2 genes expression in CCs and GCs (Fig. 5C). However, glucose uptake was significantly lower in oPOI GCs cells (Fig. 5D, *p* < 0.001).

Analysis of the first step of glycolysis showed that GLUT1 (Fig. 5E, *p* < 0.01) and HK2 (Fig. 5F, *p* < 0.05) expression and enzyme activity (Fig. 5G, *p* < 0.001) were significantly lower in CCs from oPOI patients. The quantity of active mitochondria was also decreased in CCs from oPOI patients (Fig. 5I, J) without a difference in IDH2 gene expression (Fig. 5H).

In GCs, GLUT1 expression was also significantly lower in oPOI women (Fig. 5K, *p* < 0.001). However, HK2 expression (Fig. 5L, *p* < 0.5) and enzyme activity were significantly higher (Fig. 5M, *p* < 0.001) in oPOI women than in healthy GCs. The IDH2 gene was expressed at the same level in healthy and oPOI CCs (Fig. 5N), whereas in GCs, we did not observe any changes in the quantity of active mitochondria (Fig. 5O, P).

## Discussion

In the present study, women with oPOI were characterized according to the criteria of Streuli et al., (2009) and Guzel et al., (2017) [7, 8]. The oPOI infertile women under 40 years of age may have spontaneous follicular activity, a serum AMH level ≤ 1.1 ng/mL, and a normal FSH level. The oPOI women in our study had a lower ovarian reserve, indicated as AFC, than that of healthy women. Importantly, a diminished ovarian reserve (DOR) is also defined by markers such as AMH, FSH, and AFC [17]. DOR is generally indicated by an AMH level of < 1.1 ng/mL, FSH level of > 10 IU/L, or AFC of < 5–7 total follicles [18]. In 2008, Welt et al., (2008) also suggested that POI represents different clinical states, namely occult (reduced fecundity but normal FSH levels and regular menses), biochemical (reduced fecundity, elevated FSH, and regular periods), and overt (approximately corresponding to evident POI/POF) [3]. According to these categories, the biochemical state corresponds most closely to the DOR, while occult POI corresponds to the characteristics of our group. Research from 2015 using 2 years of Society for Assisted Reproductive Technology (SART) data concluded that DOR is likely to be over-diagnosed using the SART reporting system [19]. However, the definitions of both oPOI and DOR are neither standardized nor specific [20]. As there is no evidence that DOR is a precursor to POI, we believe that DOR is distinct from oPOI. Indeed, further work is needed to determine the similarities and differences between DOR and oPOI.

In this study, we embarked on a detailed metabolic profiling of GCs and CCs to elucidate differences in mitochondrial respiration and glycolytic pathways between healthy women and those suffering from the occult form of POI. Healthy CCs have good glycolytic capacity because they need to support the oocyte metabolically by delivering pyruvate [12]. Our research confirms the glycolytic capacity of CCs from both healthy and oPOI women, but cells from oPOI women were characterized as less energetic. Similarly, the reduction of ATP production in CCs has also been reported for other reproductive disorders, such as endometriosis [21]. Another important oPOI-related change was the shift toward glycolysis in GCs with reduced ATP production. The oxidative profile of GCs emphasizes their distinct functional role within the follicle [22] however, in oPOI women, this profile was attenuated. Similar changes have been described for aging GCs, in which reduced mitochondrial respiration affects ATP production [23]. Consistent with the reduction in mitochondrial function, cells from older women with ovarian infertility utilize glycolysis more than OXPOHS to maintain ATP production [23]. In summmary, our data showed that the characteristic metabolic profiles of these cells, glycolytic for CCs and oxidative for GCs, were disturbed in oPOI women. Furthermore, our study suggests that this is a deviation from the typical metabolic roles of CCs and GCs, which may have implications for follicular health and fertility in oPOI patients.

We observed that ΔΨm was higher in both cell types from oPOI women paralleled the decrease in ATP production and mitochondrial activity. This change in the mitochondrial bioenergetic profile has previously been observed in bovine embryos exposed to elevated temperatures (41 °C), in the form of a higher ΔΨm not associated with higher ATP-linked oxygen consumptions, implying less efficient mitochondria [24]. The canonical mechanism for ΔΨm generation is via complexes I, III, and IV of the electron transport chain, which pump protons from the mitochondrial matrix into the intermembrane space. However, several studies have described an alternative mechanism for the generation of ΔΨm, namely ATP synthase running in reverse, hydrolysing ATP to ADP [25, 26]. In this context, our observation suggests that CCs and GCs have a lower ATP content, due to the hydrolysis of ATP, to maintain mitochondrial membrane potential, which highlights the importance of ΔΨm for cell welfare.

We found that these changes led to decreases in total cellular ATP concentrations in both cells. Because FF serves as a complex microenvironment for germ cell-somatic cell communication, we hypothesized that ATP levels of GCs and CCs would be reduced by glucose levels and availability in FF. Specifically, the previous study showed that FF from older women (> 40) contains less glucose than that from younger women (< 35) [27, 28]. However, we found no differences in FF glucose levels between healthy and oPOI women. Interestingly, the expression of the glucose transporter GLUT1 and the expression and activity of HK2 were lower in CCs from oPOI women. Thus, our results showed that the reduction in ATP production by CCs is due to impaired glucose uptake and metabolism. Whereas in GCs, GLUT1 expression decreased in parallel with increased HK2 expression and activity, indicating that mitochondrial activity was not affected. Therefore, an increase in hexokinase expression and activity to meet energy needs may be a compensatory mechanism. In addition, the overexpression and increased activity of hexokinase in GCs of POI women further emphasizes the shift toward glycolysis. Our results show that the metabolic changes and mitochondrial function in oPOI patients are more complicated than previously thought and are strictly dependent on the metabolic profile of cells.

Moreover, we observed that blood estradiol levels were reduced in women with oPOI, reflected by the E2/oocyte count ratio. These findings indicate potential endocrine disruption. However, no significant alterations in FSH levels were detected. Inadequate ovarian hormone secretion should contribute to a preferential increase in FSH through interactive feedback of the hypothalamic-pituitary-ovarian (HPO) axis [29], but we did not observe this in our oPOI patient population. Thus, in the case of oPOI, disruption may extend beyond the ovaries to impact the entire HPO axis.

## Conclusions

An important finding of this study was that oPOI dysfunction is only partially similar to that observed during aging, despite the fact that oPOI is often referred to as premature ovarian aging [30]. We found that GCs in particular use metabolic plasticity to enable their survival. However, these changes affect cell functionality, despite the compensatory mechanism associated with upregulation of hexokinase activity. In addition, we found that decreased glucose transporter and hexokinase activities contributed to lower ATP production by CCs from oPOI patients. These observed changes were all accompanied by a reduction in the number of high-quality blastocysts and clinical pregnancies in oPOI women undergoing IVF. Also, these results suggest that oPOI-associated changes in hormone homeostasis are not limited to ovarian dysfunction.

Table 1 legend: oPOI - occult premature ovarian insufficiency; BMI – Body mass index [kg/m2]; AMH - anti-Müllerian hormone [ng/ml]; FSH - follicle-stimulating hormone; E_2_ – estradiol [pg/ml]; AFC – antral follicle count; MII – oocytes in MII stage; MI – oocytes in MI stage; ICSI – Intracellular Sperm Injection; RO - retrieved oocytes; GQB – good quality blastocysts; GB rate – good blastocyst rate; CP rate – clinical pregnancy rate.

## Data Availability

The experimental data and the simulation results that support the findings of this study are available in Jagiellonian University RODBUK repository [https://rodbuk.pl/].

## Abbreviations

AFC: Antral follicle count
AMH: Anti–Müllerian hormone [ng/ml]
BMI: Body mass index [kg/m2]
CCs: Cumulus cells
CP rate: Clinical pregnancy rate
DOR: Diminished ovarian reserve
E_2_: Estradiol [pg/ml]
FF: Follicular fluid
FSH: Follicle–stimulating hormone
GB rate: Good blastocysts rate
GCs: Granulosa cells
GLUT1: Glucose transporter 1
GLUT4: Glucose transporter 4
GQB: Good quality blastocysts
HK2: Hexokinase 2
ICSI: Intracellular Sperm Injection
IDH2: Isocitrate dehydrogenase (NADP(+)) 2
IVF: In vitro fertilization
MI: Oocytes in MI stage
MII: Oocytes in MII stage
oPOI: Occult premature ovarian insufficiency
OXPHOS: Oxidative phosphorylation
POF: Premature ovarian failure
POI: Premature ovarian insufficiency
POI: Primary ovarian insufficiency
RO: Retrieved oocytes
RT: qPCR–real–time quantitative polymerase chain reaction
SD: Standard deviation
SLC5A1: Solute carrier family 5 member 1
SLC5A2: Solute carrier family 5 member 2
TCA: Tricarboxylic acid cycle
